# Visions of Artificial Intelligence and Robots in Science Fiction: a computational analysis

**DOI:** 10.1007/s12369-022-00876-z

**Published:** 2022-07-18

**Authors:** Hirotaka Osawa, Dohjin Miyamoto, Satoshi Hase, Reina Saijo, Kentaro Fukuchi, Yoichiro Miyake

**Affiliations:** University of Tsukuba, Tsukuba, Japan

## Abstract

Driven by the rapid development of artificial intelligence (AI) and anthropomorphic robotic systems, the various possibilities and risks of such technologies have become a topic of urgent discussion. Although science fiction (SF) works are often cited as references for visions of future developments, this framework of discourse may not be appropriate for serious discussions owing to technical inaccuracies resulting from its reliance on entertainment media. However, these science fiction works could help researchers understand how people might react to new AI and robotic systems. Hence, classifying depictions of artificial intelligence in science fiction may be expected to help researchers to communicate more clearly by identifying science fiction elements to which their works may be similar or dissimilar. In this study, we analyzed depictions of artificial intelligence in SF together with expert critics and writers. First, 115 AI systems described in SF were selected based on three criteria, including diversity of intelligence, social aspects, and extension of human intelligence. Nine elements representing their characteristics were analyzed using clustering and principal component analysis. The results suggest the prevalence of four distinctive categories, including human-like characters, intelligent machines, helpers such as vehicles and equipment, and infrastructure, which may be mapped to a two-dimensional space with axes representing intelligence and humanity. This research contributes to the public relations of AI and robotic technologies by analyzing shared imaginative visions of AI in society based on SF works.

## Introduction

Science fiction (SF) as a literary genre draws on the interaction between technology and society. SF describes the influence of technology on society in terms of human drama based on compelling storytelling. The SF genre is an important component of our contemporary society owing to its popularization of science and technology along with an understanding of their transformative potential. The impact of SF is significant in both academic and industrial fields. For example, iRobot, manufacturer of the Roomba brand of cleaning robots, named their company based on Isaac Asimov’s novel “I, Robot.” Palmer Luckey, who founded the VR headset company Oculus, expressed influence of Neal Stephenson’s “Snow Crash”, Ernes Cline’s “Ready Player One”, and Reki Kawahara’s “Sword Art Online” on his work. Researchers in academic fields including information science, mechanics, robotics, and artificial intelligence (AI) are also typically broadly familiar with SF. For example, Nature Publishing Group has organized a series of short SF stories in “Nature” magazine since 2009. These stories help researchers in many different disciplines and the general public understand visions of future technologies more easily. Along these lines, Microsoft commissioned short stories from several SF writers based on their laboratory technology. The short stories were organized as a free anthology titled “Future Visions”. In China, international SF conferences are often actively supported by government organs. In Japan, the Society of Artificial Intelligence, the Japanese Society for Robotics, the Society for Automatic Measurement and Control, the Society of Human Interface, and other academic societies related to information, machinery, and electricity have continuously created special articles on SF. An increasing number of academic organizations also specialize in SF, such as the SF Film Institute on HCD-Net. Many researchers have attested as to the influence of SF on their work, and many technical terms are derived from SF, including “robot,” “robotics,” “technical singularity,” and “cyberspace.”

Science fiction is widely understood to have motivated a broad variety of research and development. Kurosu 2014; Marcus et al. 1999; Mubin et al. 2016; Nagy et al. 2018; Schmitz et al. 2008; Tanenbaum, Tanenbaum, and Wakkary 2012; Troiano, Tiab, and Lim 2016). From a more positive perspective, there are several notable examples in which SF writers have participated in various projects as technical advisors. Science fiction writers such as Bruce Sterling and Cory Doctorow are frequently involved in conferences and policy decisions on information technology (Sterling 2009). Satoshi Hase and Taiyo Fujii have participated in the ethics committee of the Japanese Society for Artificial Intelligence and are involved in the creation of ethical standards, and the Japanese Writer’s Community also cooperated with a survey (Ema et al. 2016). Liu Cixin, the author of “The Three-Body Problem”, joined a Chinese company (Cixin, Nahm, and Ascher 2013). The acceptance of AI and robotic anthropomorphic systems in society has been a major theme in SF for many years. Various concepts have been generated in the interaction of both fields, from Isaac Asimov’s Three Laws of Robotics (Asimov 1950) (McCauley 2007) to Verner Vinge’s technological singularity (Singularity) (Vinge 1993). For example, Isaac Asimov’s SF stories exploring robotics as a theme have been discussed as a future vision of humans and AI. His Three Laws of Robotics (Asimov 1950; McCauley 2007) are referred to in the Chiba University Robot Charter (Matsuo 2017) and Korea’s Robot Ethics Charter (Shaw-Garlock 2009). Moreover, SF has always exerted a significant influence on the development of AI technologies. Shedroff et al. defined four ways in which SF influences designers and researchers, including through (1) inspiration, (2) by establishing expectations, (3) by creating a social context, and (4) by describing new paradigms (Shedroff and Noessel 2012). SF has also been used as a teaching method for AI ethics (Burton et al. 2018).

While SF stories and images have helped to envision the future, fictional depictions to involve some important constraints. First, SF stories are generally produced for the primary purpose of entertainment rather than as a scientific investigation of the possibilities of future societies. There are also concerns as to the dark visions depicted by some SF media, which sometimes involve themes such as those of robotic systems replacing humans or going catastrophically out of control. Although SF writers are professional storytellers that rely on themes involving science and technology in their work, they are not typically science and technology professionals. Hence, there is some risk that the narrative logic inherent in SF may neglect the context of real social situations. For example, the AI referred to as Skynet appearing in the Terminator franchise is occasionally referenced as a negative vision of AI in the technical literature (Mubin et al. 2016). In addition, there are works that do substantially involve social problems that may exist in the background of future societies. Owing to recent rapid development of AI, the ethical problems posed by the use of such technology in society have been discussed as a practical matter in various contexts. As a result, caution should be exercised in applying ideas from classic SF, including visions of intelligent anthropomorphic robotic systems to real-world problems directly. Out-of-context applications of science fiction ideas have also been criticized. For example, Jean-Gabriel Ganascia says that various technologies have been overhyped as a result of an abuse of the term *technical singularity* by Ray Kurzweil (Ganascia 2010, 2017). A humanities scholar, Jennifer Robertson, described the vision of the future depicted by the Japanese government as problematic, stating that it tended to confirm sexist representations inherited from the classic SF works (Robertson 2011). Given that some scientists and technicians have used ideas from SF unscrupulously in scientific communications, researchers must attend to the possibility that the deliverables of such advanced technologies envisioned in fiction may not be compatible with society, or that some implementations may be impractical, unethical, or ill-advisedi.e., Skynet, etc. While the scenarios presented in SF have the advantage of helping depict the future, they are limited by their fictional nature. In addition, in some works, it is important to consider the social problems and conditions that contributed to the background of the art, rather than the specifics of fictional techniques. The application of SF thus requires careful attention, as such works were necessarily created within the context of a specific time period. Hence, fictional ideas should not be uncritically adopted as a basis for future developments without an appropriate consideration of the context of such works.

These examples suggest that science fiction can help us understand how the public imagines future AI and robots, as opposed to directly predicting the future. Science fiction stories and the new technologies they describe provide good indicators of how the general public perceives technology. Therefore, by analyzing AI and robots depicted in existing science fiction works, the general reception of new technologies developed by researchers and engineers may be more effectively predicted. In this study, we analyzed popular preconceptions of AI and robotic systems by investigating depictions of such technologies in existing science fiction.

This study surveyed depictions of AI and robotic systems in SF with the help of SF experts to analyze types of stereotypes applied to anthropomorphic systems and visions of their development and adoption in SF. The remainder of this study is organized as follows. Section 2 explains the background of the relationship between science fiction and science and technology, including artificial intelligence. Section 3 explains how we determined the SF criteria to avoid arbitrary selection. Section 4 explains the statistical methods used to perform the data analysis. Section 5 discusses the stereotypes and visions exhibited by these works in the SF genre. The contribution and limitations of the present work are described in Sect. 6, and Sect. 7 presents our final conclusions along with some possible avenues for future research.

## Background

### The impact of Science Fiction: speculative inferences of Social Development from Scientific reasoning

SF is a literary genre centered on stories developed based on themes relating to various types of science, technology, or scientific methods. The definition of modern SF is significantly broader than that of the content that was originally called SF. The conventional definition of the genre is imprecise, varying by author, critic, and reader and is often controversial (Tatsumi 2000). The concept of SF, in terms of stories based on scientific thinking, has a long history. However, such genres attracted increasing attention owing to the development of science in the context of the Industrial Revolution. For example, in Bram Stoker’s ”Dracula”, characters try to save a person who was attacked by a vampire via a blood transfusion. The work itself is a horror novel, but such literary techniques have been widely used in horror, action, and drama genres. Therefore, such works can be regarded as having some overlap with SF. Overall, SF is heavily influenced by science and technology in the fields of physics, chemistry, biology, space engineering, mechanical engineering, electrical engineering, and information technology.

There are several reasons for the widespread popularity of SF. For example, owing to the development of science and technology, there are many well-known cases in which conventional scientific knowledge was overturned by groundbreaking research. Typically classic SF works have rarely addressed science and technology in a rigorous or truly scientific manner. However, ideas of sci-fi technologies envisioned by these works, such as robots and space travel, have been maintained over several generations and widely explored in the genre. Often, the scientific framework of such stories may be improved over time to reflect changing contemporary ideas, so such radical possibilities are often explored usefully in SF despite its typical lack of true scientific rigor and process. For example, SF stories based on time machines or faster-than-light navigation can be considered as such works. SF has explored a wide variety.

of conceptually conceivable worlds, such as planets or universes with different physical laws. The plausibility of such descriptions cannot be easily assessed, even if though such speculative descriptions may be based on patterns derived from scientific inference. Many sci-fi works are based settings that diverge dramatically from the real world, such as fictional worlds in which the speed of light is extremely slow, stories that unfold under high gravity (Robert Forward ‘dragon’s egg’), or works that explore the concept of planetary intelligences (Stanislaw Lem’s Solaris’). Furthermore, there are examples in which the reactions of society to new technologies are realistic, though the presented technologies or scientific advancements themselves may be fictional. For example, Sakyo Komatsu’s “Virus” described a pandemic that decreased the population of society through a depiction of characters onboard a train. His explanation is referred to as having predicted the COVID-19 pandemic in Japan, even though the virus in the story is fictional (Omori 2020). Hence, we can consider that SF works may sometimes accurately predict future events or scenarios. For example, although the specific technologies of the Internet itself were not directly predicted, novels which foresee a world connected by the communication networks have a long history. For example, Shinichi Hoshi wrote ‘Voice Net’, which featured an AI-based intra-net service based on telephone networks. Some works focus on portraying human beings and society through fictional technology.

In this paper, we define SF as genre of stories that depict the imaginative settings and the reactions of people in fictional societies, with themes involving scientific techniques and reasoning. It includes stories based on technologies that may not necessarily be accurate according to current scientific knowledge or have not yet been achieved.

### How AI and robotic Systems are portrayed in Science Fiction: Social Agents and Human Extension

Artificial intelligence and robots are often portrayed in SF as social agents or technologies that extend human capabilities. Several depictions of artificial slave appear in classic stories. For example, golems in Jewish folklore might be considered as a representative example of an animated construct. Golems are anthropomorphic beings that can be controlled by a human, in a manner somewhat analogous to that of a computational agent. Similar to stories involving robots, these stories often involve a theme of golems breaking free of human control or escaping. Mary Shelley’s “Frankenstein” is widely known as a classic work that may be considered as a predecessor of later SF, which tells the story of a monster created by Dr. Frankenstein by stitching together dead and dismembered bodies, which then escapes and kills Frankenstein’s family and friends in revenge for his unfortunate creation. Karel Capek’s ”R.U.R.” is a story about artificial agents that revolt against their creators. The robots depicted in this work are not mechanical artifacts, but rather biological workers created via technology. Similarly, his work “War with the Newts” does not deal with artificial intelligence itself, but it does detail the consequences of human training of intelligent salamanders on which society depends, and a revolt is foreseen. There are several works on the controllability of artifacts, which consider new technologies and their social impact. Stories about robots have often centered on the theme of fear of artificial creations going out of control. Several reasons for this revolt have been explored, but one common criteria of such story is that events cannot be foreseen beforehand.

These fears are called the Frankenstein Complex, after Frankenstein’s monster (Mccauley and Hall 2007). Concerned about the tendency to equate artifacts with monsters, Isaac Asimov, a prominent classic SF writer, introduced the Three Laws of Robotics in his work ‘I, robot.‘ (Asimov 1978). In many of Asmiov’s works, robots function as autonomous artifacts programmed to adhere to the following principles in order of priority. 1. A robot may not injure a human being or, through inaction, allow a human being to come to harm. 2. A robot must obey the orders given it by human beings except where such orders would conflict with the First Law. 3. A robot must protect its own existence as long as such protection does not conflict with the First or Second Laws. The Three Principles of Robotics were used repeatedly and extensively in Asimov’s own later works, and are known to have greatly influenced many other authors. There have also been proposals, such as Chiba University’s ‘Chiba University Robot Charter’ and Korea’s Robot Ethics Charter, to establish actual control codes based on Asimov’s three laws (Matsuo 2017; Shaw-Garlock 2009). However, it should be noted that these three principles are merely a narrative device. In fact most of the short stories comprising ”I, Robot.” are centered on interactions between humans and constructs that cannot be predicted solely based on the Three Laws. There are many more examples of similar literary themes in which an artifact seeks or gains a human soul. For example, “The Adventures of Pinocchio”, a children’s story by Carlo Collodi in 1883, also describes examples of intentional behavior by artifacts. Many stories focus on the of nonhumans entities obtaining intelligence or souls similar to those of humans. For example, the Greek myth of Pygmalion involves a sculpture of a woman that behaves like a human being and marries the sculptor who created her(Kaplan 2004). In many of these narrative forms, artificial intelligence questions the nature of intelligence itself. Barrington Bayley’s “Soul of the Robot” tells the story by a robot in the first person, and incorporates some material on artificial intelligence material, including the frame problem. However, in essence the work describes how the robot protagonist’s autonomy is a result of human intelligence. The theme of women’s souls or autonomy being limited or controlled by men has been portrayed in Western literature alongside critical investigations of sex and gender divergence. Amy Thomson’s ”Virtual Girl” presents a critical exploration of these themes from the perspective of a female robot.

Human augmentation through information technology is another common theme in SF works on artificial intelligence and robotics. For example, the impact of VR and HCI has been frequently explored in cyberpunk SF. Cyberpunk emerged as a trend in SF in the 1980s. The genre often presuppose a futures in which the bodies or minds of humans are augmented with technological systems. Alice Bradley Sheldon, better known by her pen name James Tiptree, Jr., was an early writer of cyberpunk SF who explored the idea of a woman who remotely controls a mindless but living artificially constructed separate body as an advertisement for a corporation in “ The Girl Who Was Plugged In”. The idea that technology can compensate for basic disparities such as gender was later inherited by Donna Haraway’s “Cyborg Manifesto” and the associated movement (Haraway 2000). Similarly, the artist Sputniko!, who creates art to overcome gender differences with technology, stated that her work “Crowbot Jenny” was influenced by Donna Haraway. Many SF works discuss the theme of transforming a person into a superhuman by expanding their intelligence or changing their values. Writers such as William Gibson and Bruce Sterling have contributed to this trend. Jun Rekimoto was influenced by this idea as an HCI researcher at SONY CSL/Tokyo University. For example, JackIn, a remote presence technology that seamlessly superimposes a users’ body on remote viewpoints, was named after a phrase from William Gibson’s Neuromancer (Kasahara et al. 2017). Augmented Human, as he put it, was based on the expansive ideas of human nature proposed by cyberpunks (Rekimoto 2014). Masahiko Inami, a VR researcher, likewise pointed out the influence of cyberpunk SF on research, stating that the use of retroreflective materials for transparency (Inami, Kawakami, and Tachi 2003) was influenced by optical camouflage depicted in the cyberpunk SF “Ghost in the Shell”. In Superhuman Sports, of which he is an advocate, this extension of humanity has been tested in other ways (Orikasa et al. 2017). Post-cyberpunk SF is often seen as a positive indicator of this orientation. For example, Verner Vinge, an advocate of the idea of a technological singularity, proposed in his work that the intelligence tends to extend itself, and defined the singularity in terms of such extension, without making a fundamental distinction between human and machine. This concept is sometimes called intelligence amplification (IA) in comparison with AI (Leinweber 2009). Greg Egan used his knowledge of physics and cognitive science to actively describe changes in humanity as an author (Nichols, Smith, and Miller 2007). In addition, stories focused on one Internet technology and social media networks, which are relatively novel developments in society that augment human capabilities, are considered to be included in these works. Dave Eggers’ “The Circle,” for example, depicts the consequences of a world built on corporate social network approval, with each technology being presented as a realistic manifestation of future concern.

## Designing an analysis of AI in Science Fiction

### Making Criteria for Review

Following previous studies (Mubin et al. 2016; Reeves 2012), we first established a set of review criteria to avoid an arbitrary survey. Previous research on the use of robots in SF suggests the importance of selecting works based on unified criteria (Mubin et al. 2019). Hence, we used the Science Fiction Hall of Fame as a specific organization to limit the scope of the SF literature review. However, representations of AI in SF are more diverse than those of robots, and thus simply following existing standards was difficult. It was also difficult to conduct cleanly separated surveys of robots and artificial intelligence. Intelligent information processing technologies that emerged before the name artificial intelligence existed were often referred to as robots. Both robots without physical bodies and artificial intelligence with physical bodies have appeared, so narrowing the range of the two terms was not useful. Importantly, AI in earlier SF works are generally not labeled as “AI” as they were written before the definition of AI was established; hence, it was not possible to collect such works simply by searching for the word.

To establish the review criteria, we conducted online discussions with 15 experts from the organization Science Fiction and Fantasy Writers of Japan and selected seven experts, including six critics and one writer, with different specialties in foreign and domestic SF works, including comics, young adult novels, visual works, and drama. This writer’s association is founded on 57 years ago, and includes authors, critics, translators, and researchers. It is widely considered most authoritative association for the study of Sci-Fi. Therefore, we selected the organization as the partners in this study. Based on a half-day face-to-face discussion between them and ourselves (a scientist, two engineers, and a philosopher), we established the following criteria to select AI systems portrayed in SF stories.

In the prior discussion, including the above review of the literature, we identified three different roles for AI technology described in SF stories.

- Stories considering the possibilities of alien intelligence. These depict different forms of intelligence such as programs, robots, and extraterrestrial intelligence. Stevelts, the group intelligence of nanomachines in Greg Egan’s “Steve Fever,” was mentioned in the discussion.
- Stories considering aspect of social intelligence. Even if a detailed implementation of intelligence is not described in the story, this category included works focused on social interactions with AI. In the discussion, Bokko-chan from Shinichi Hoshi’s “Bokko-chan,” a parrot toss response robot, was mentioned.
- Stories considering the possibility of artificial extension of human intelligence. The theme of this category was the expansion of human cognitive ability through advanced interfaces between humans and robots or machines, augmented humans, the internet, and social networks. In the discussion, Chohei Kanbayashi’s “Yukikaze” was cited as an AI for a combat aircraft designed to extends the operator’s ability.

We collected stores on artificial intelligence and robotic technologies from science fiction on as broad a basis as possible with the cooperation of experts. Therefore, it was necessary to collect AI and robots that appeared in the works by setting a broad standard including as wide a range of intelligence technologies as possible to cover the diversity of the subject. This is reflected in the first policy on the diversity of intelligence. Artificial intelligence and robotics in science fiction are applicable to social agents or extensions of human intelligence. This background is explained in Sect. 2.2, and the criteria from this aspect are reflected in policies 2 and 3.

The information used to classify AI in the selected SF using the above criteria was examined, as shown in Table 1. Considering the characteristics of AI that are important in literature and those that are important in terms of AI technology, the following 20 factors and work summaries were collected. In addition, we obtained an overview of each story to verify the correctness of the factor.

**Table 1:** Collected AI factors (11 factors shown in gray were quantified and normalized after data collection in Sect. 2.2)

AI name	The name of the AI
Work name	The name of the work in which AI appears
Year	The year in which it was first published
Media	First media to appear (novel, comic, movie, and play)
Country	First published country
Friendliness	How AI is friendly to humans
Generality	Versatility of AI capabilities
Consciousness	How conscious AI is
Crowd	How many AI groups are there to act together?
Network	How much AI is connected to the network
Language	Linguistic abilities of AI
Learning	AI’s ability to learn something
Physical	How much physical reality does AI have?
Human-shape	How does AI look like a person?
Maker	How many people (organizations) associated for AI creation
Independence	How independent AI is from humans
Task	Tasks performed by AI
Communication	Way AI communicates
Material	AI materials
Energy	Energy sources for AI

First, we designed the following review priorities for the survey requested of SF experts. These three characteristics have been cited as the impact of AI in science fiction on readers.

- Diversity. The age of the publication and the media in which the work was published must not be biased toward a specific field.
- Impact. Works with a significant social impact should be included. Those with less social impact but unique characteristics were also appropriate for collection.
- Uniqueness. In the case of AI with similar characteristics, the original work was included inserted. When multiple AI systems appear in a single work, the system with the most unique features was collected.

### Collecting Data

We collected 115 portrayals of AI from experts after they performed mutual quality check. The average year of publication of the works collected was 1981 (with a standard deviation (SD) of 26.8 years). The oldest character was the human cyborg described in “Rakouské celní úřady” written by Jaroslav Hašek in 1912, and the newest was Girl M, a bionic AI controlled by slime mold, in “Long Dreaming Day,” written by Katsuie Shibata in 2019. Among the works collected, 55 were published in Japan, 52 in the US, three in the UK, two in Poland, and one in the Czech Republic, with two works being published worldwide simultaneously. 93 were first released as novels, 12 as comics, seven as movies (two of which were animations), and three as plays. Fourteen works were published before 1945, 64 works were published after World War II until 1995, before the widespread adoption of the Internet, and 37 works were published after 1995. We confirmed that the distribution had sufficient diversity in each decade, as shown in Table 2.

**Table 2:** Each factor in four clusters (* An asterisk indicates that a significant difference of *p* < .05 was obtained on Tukey’s test.)

	Machine /M	Human /H	Buddy /B	Infrastructure /I
Friendliness	0.58 (SD 0.43)	0.84 (SD 0.25)	0.56 (SD 0.33)	0.82 (SD 0.33)
Generality *M-H, M-B, H-I, B-I	0.04 (SD 0.13)	0.41 (SD 0.32)	0.34 (SD 0.45)	0.79 (SD 0.26)
Consciousness *M-H, M-B, M-I	0.08 (SD 0.26)	0.85 (SD 0.31)	0.84 (SD 0.27)	0.81 (SD 0.38)
Crowd *M-H	0.28 (SD 0.46)	0.01 (SD 0.07)	0.11 (SD 0.27)	0.16 (SD 0.34)
Network *M-H,M-B,M-I	0.11 (SD 0.32)	0.07 (SD 0.18)	0.02 (SD 0.10)	0.98 (SD 0.07)
Language *M-H, M-B,M-I	0.08 (SD 0.19)	0.97 (SD 0.12)	0.74 (SD 0.32)	0.95 (SD 0.13)
Learning *M-H,M-B, M-I	0.15 (SD 0.35)	0.56 (SD 0.43)	0.57 (SD 0.45)	0.87 (SD 0.29)
Physical *M-I, H-I, B-I	0.94 (SD 0.13)	0.97 (SD 0.11)	0.93 (SD 0.15)	0.61 (SD 0.40)
Human-shape *M-B, I-B, I-H, M-H, M-I	0.17 (SD 0.27)	0.89 (SD 0.22)	0.06 (SD 0.22)	0.43 (SD 0.44)
Maker	0.17 (SD 0.34)	0.22 (SD 0.27)	0.15 (SD 0.27)	0.24 (SD 0.36)
Independence	0.89 (SD 0.27)	0.95 (SD 0.22)	0.94 (SD 0.22)	0.87 (SD 0.33)

Here, we discuss 20 factors. We first estimated that 11 factors were quantifiable, including maker and independence factors. However, deeper discussion revealed that the latter two factors were not appropriate to scalar value (darker gray shading). We then selected nine factors, as displayed in the light gray cells in Table 1, for normalization. The participants were divided into five steps using two experts. For example, in the case of an animal-type AI, the human-type value was 0.25, and in the case of an AI with a part such as a neck or a hand, the human-type value was 0.75. The Cohen’s Kappa value of two experts results was 0.86 (> 0.8) and we estimated that the collected data were of sufficient accuracy.

## Analysis

**Fig. 1:**
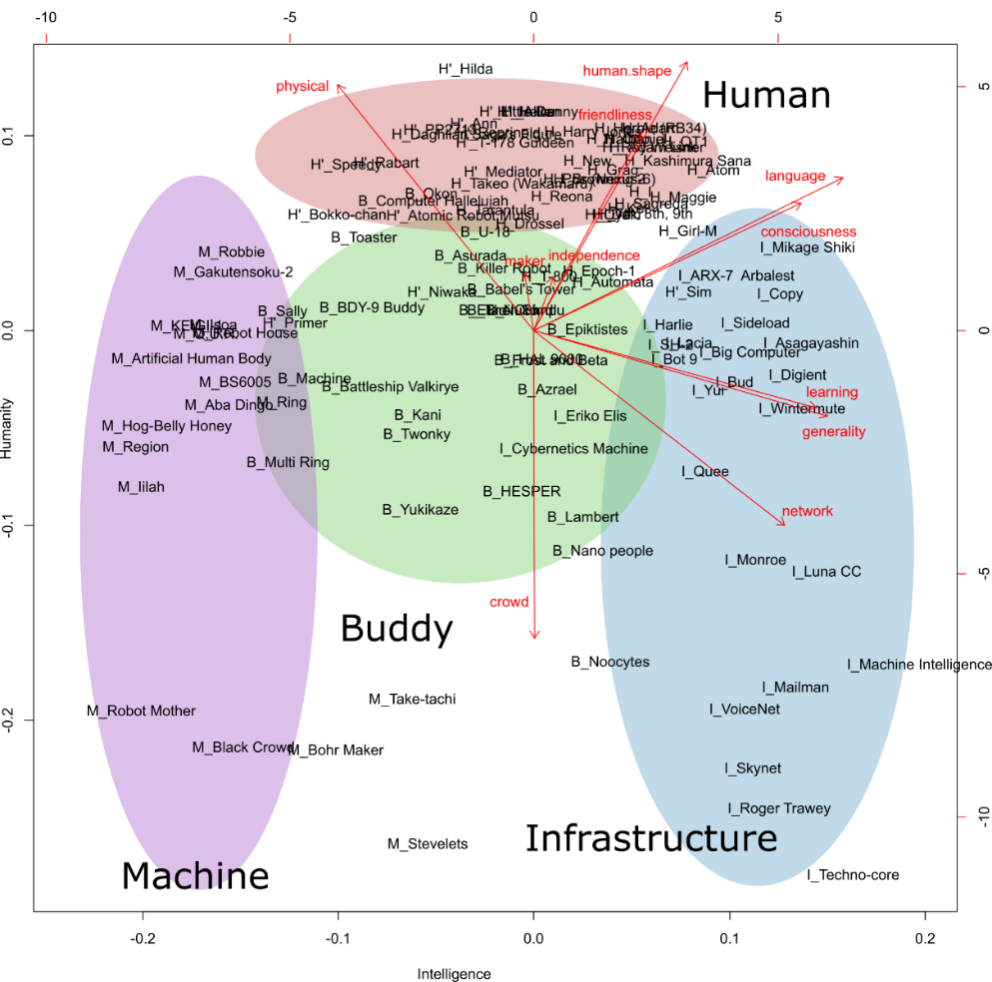
AI in SF on a PCA Map (each AI name has a label in front of the name)

We used principal component analysis (PCA) to extract the main factors from multiple factors. As a result, of 43.3% the information in the second main component and 64.4% in the fourth main component was extracted. We decided that up to the fourth main component represented our data well. The first axis (24.4%) with a high contribution ratio was labeled *intelligence*, whereas the second axis (19.0%) was labeled *humanity*, which suggests familiarity with human society. The third axis (12.0%) with a high contribution ratio of independence was labeled as *independence*, and the fourth axis (9.1%) with a high maker factor contribution ratio was labeled *maker*. Hierarchical cluster analysis was also performed for the 11 factors. The distance between each element was measured in terms of the Euclidean distance and classified using Ward’s method. The cluster tree was divided into four clusters based on the distance between 10 distinctive points. These four clusters were characteristically separated on a PCA map constructed with the first (intelligence) and second (humanity) factors as primary axes, as Fig. 1 is mainly constructed by nine factors. There was no distinctive clustering in the PCA map constructed by the third (independence) and fourth (maker) factors.

Based on the characteristics of the four clusters, we labeled them as Buddy, Machine, Infrastructure, and Human. As shown in Fig. 1 (arrows), several factors exhibited mutual relationships. For example, both the pairing of human shape with friendliness and consciousness with language ability contributed to increased intelligence and humanity, as shown in Fig. 1. Increasing generality, learning, and higher network connectivity contributed to increased intelligence, but also decreased humanity. The crowd factor did not contribute to intelligence, but did contribute to decreased humanity. Physical factors contributed to increased humanity but also decreased intelligence. Independence and maker did not contribute to either axis Electricity was the most common energy source, and other sources were diverse, with no significant trends.

The average values of the axes for each cluster are given in Table 3. The combinations that showed significant differences on Tukey’s multiple comparison test are listed in the first table cell. The distribution by year of publication is shown in Table 2. These averages exhibit a weak tendency in which Machine and Human type AIs are typically slightly older archetypes than Buddy and Infrastructure. In addition, it is remarkable that all works with Infrastrcuture-type AIs were written after 1970 (The year of publication of “Voice Net,” written by Shin’ichi Hoshi). This suggests that most of this AI type was generated after computer and telecommunication technologies were developed.

**Table 3:** Four clusters with years

	Machine	Human	Buddy	Infrastructure	Total
1912–1945	3	7	2	0	12
1946–1995	9	32	19	14	74
1996–2019	6	7	5	11	29
Total	18	46	26	25	115
Average	1977.5 SD 29.2	1979.3 SD 21.9	1981.4 SD 21.8	1994 SD 18.0	1981.1 SD 27.0

### Machine-type AI

The cluster was populated by AI that are shown as being less intelligent than humans (as regarded by human beings). In contrast to the human type, this type is unique in that it exhibited low generality (0.04 (SD 0.13)), low consciousness (0.08 (SD 0.26)), low language ability (0.08 (SD 0.19)), and low learning ability (0.15 (SD. 35)), and low human-shape (0.17 (SD 0.27)). Representative examples of this type include the Robot Mother in “The Mechanical Mice” by Maurice Hugi, “Hog-Belly Honey” by R.A Lafferty, and KEIGI-1 in “Inter Ice Age 4” The tasks performed by AI range from babysitting to weapons, but many of them do not learn from the environment. These appear in stories as unintelligent automated machines for solving specific problems. Alternatively, these machines are provided in an environment in which the protagonists cannot interfere. Their inflexibility often damages human society.

### Human type AI

Human-type AI was the most common of the four types (40%). From Table 2, it may be observed that this type of AI has been depicted in SF from the beginning to the present day in every decade. Specificities in this type included moderate generality (0.41 (SD 0.32)), high consciousness (0.85 (SD 0.31)), high language skills (0.97 (SD 0.12)), moderate learning skills (0.56 (SD 0.43)), high physical appearance (0.97 (SD 0.11)), and high human-shape (0.89 (SD 0.22)). Representative examples of this type include Atom in Osamu Tezuka’s “Astro Boy” and several robots in Isaac Asimov’s “I, Robot”. This type is thought to include the traditional SF theme of artificial humans taking the place of humans. Regarding the tasks performed by the AI, housework was the most common task (11 cases), followed by outdoor physical labor (5 cases) follows. Human-type AI act independently as members of society, learn from their environment, and perform general tasks, just as humans do. In general, most of these characters are treated as a metaphor for humans. This type of AI was concentrated in a relatively small cluster, as illustrated in Fig. 1. This seems to be because the human image was the norm for these characters.

### Buddy-type AI

The buddy-type AI were identified as human-dependent, conscious, and collaborative agents that typically helped with work. They are similar to the human type, but distinct in terms of their low generality (0.24 (SD. 45)), slightly lower language skills (0.74 (SD 0.32)), and low human-shape (0.06 (SD 0.22)). Representative examples of this type include HAL 9000 (a spaceship AI) in “2001: A Space Odyssey” by Arthur C. Clark, Yukikaze (a combat aircraft) in “Yukikaze” by Chohei Kanbayashi, and Asurada (a semiautomatic car) in “Future GPX Cyber Formula” by Mitsuo Fukuda. The buddy-type AI typically exhibits a tool-type shape such as that of a vehicle, performs specific tasks for each tool, and sometimes accepts commands via non-verbal input. Among the tasks performed by the AI, the most common task was military (eight cases), followed by automatic operation (four cases). The buddy type of AI works with humans to extend their cognitive abilities. These AI’s unique consciousness (autonomy) interferes with and effects human consciousness. There are also cases in which they run out of control owing to dilemmas involving human orders.

### Infrastructure-type AI

Infrastructure-type AI are often less physically active, include substantial network connectivity and language capabilities, and are often used as social infrastructure. In contrast to the human type, this type is characterized by high network connectivity (0.98 (SD.07)), slightly lower physical appearance (0.61 (SD 0.40)), and moderate human-shape (0.43 (SD 0.44)). Representative examples of this type include Skynet in “Terminator” by James Cameron, Wintermute in “Neuromancer” by William Gibson, and Lacia in “Beatless” by Satoshi Hase. The most common task performed by the AI was facility management (15 cases). The image of infrastructure-type AI is thought to have been created mainly after World War II with the development of computer and communication technologies. The average year of publication was 1994 (SD 18.0), as shown in Table 2, which was more recent than the other categories. The implementation of computer networks has varied over time, and some works have been represented as AI over telephone networks (“Voice Net” by Shin’ichi Hoshi). In “Voice Net,“ a recommendation system and evaluation economy similar to that of the Internet society is achieved by an AI system composed of telephone networks installed in buildings.

## Discussion of SF stereotypes and possibilities

### AI factors that imply contributions to intelligence and humanity

In order to explain the developed framework for AI and robot systems, we identified some factors suggestive of intelligence and humanity in fictional AI. Our analysis of fiction, shown in the PCA map ( Fig. 1), contributed to revealing these hidden relationships.

Embodiment is an important factor in the field of AI (Brooks 1991). From an SF viewpoint, it has been considered as a factor contributing to humanity in terms of familiarity with human society. However, our analysis suggests that embodiment exhibits a two-sided influence on how intelligence influences people. If a humanlike shape is attributes AI, portrays typically involved increased intelligence. However, general physical attribution contributed to decreased intelligence. This tendency was the same as that suggested in the robotics design and human-computer interaction (HCI) design principle called the adaptation gap (Komatsu, Kurosawa, and Yamada 2012), in which humanlike attributes contribute to more intelligence.

Language ability, consciousness, learning ability, generality, and network connection exhibited similar tendencies in contributing to an increase in portrayed intelligence. However, their contributions to humanity were slightly different. The language ability of AI and their consciousness showed the same tendency to weakly increase humanity. In contrast, learning ability and generality contributed weakly to decreased humanity, and network connection marginally contributed to decreased humanity. It may be useful to explain language capabilities rather than the versatility of AI to convey the advantage of AI to non-experts.

It is also remarkable that the crowd factor simply contributes to decreased humanity. It is difficult for people to imagine an intelligence comprised of many less complex intelligences. Hence, care should be taken to communicate the harmlessness of this kind of artificial crowd intelligence.

### Avoiding stereotypes: human and machine

Human-type AI in SF seems to have been used as a motif for humans from different cultures, and machine-type AI in SF seems to have been used as a motif for an uncontrollable machines, with each being a stereotypical aspect. Human-type agents, like Karel Capek’s R.U.R. which coined the term “robot,” are reflective of racial discrimination and slave labor engaged in housework and labor. This is thought to have functioned in the narratives to describe aspects of the coexistence of different human beings in society such as fear.

Machine-type AI are another stereotype in SF, representing the theme of machines that work independently and may cause problems by going out of control. The fear of this type of uncontrollable machine goes back to the stories of golems. The main features of this type of AI are its apparent lack of intelligence and inflexibility.

Although we acknowledge that these AI images work well in the literature, we are concerned that they may not present a technically realistic image. We are also concerned that these fictional works may induce stereotypes of AI. Although creating human-like intelligence is a primary goal of AI, today’s AI are not as intelligent as humans, but they are also not necessarily unsophisticated machines regulated by simple rules easily understood by humans. Human-like images are often used, especially in the design and promotion of commercialized AI; however, we think researchers and technologists should take care to avoid the overuse of this image, and should explain their technologies to avoid these stereotypes.

### Beyond Anthropomorphism: non-human buddies and social infrastructure

We believe that buddy-type and infrastructure-type AI will be more important in communicating the vision of future AI designs. Buddy-type AIs are not like a human, but they performs tasks in cooperation with humans. A Buddy-type AI’s work deals with the problem of how to compromise between AI and human decision-making as a unified working system, including issues such as the division of roles between humans and AI in automated driving. These examples can shed light on how the coupling of humans and AI my function under extreme conditions. Yukikaze is a typical example. It depicts the process in which a human pilot interacts with a heterogeneous helper intelligence, and in the process, accepts decisions and makes heterogeneous decisions. This is a challenge that needs to be addressed when dealing with autonomous weapons, autonomous vehicles, and other similar issues.

The infrastructure type is a new image that appeared alongside the development of the computer. These systems were imagined as information technology progressively developed. For example, “Beatless” by Satoshi Hase depicts a world of AI after a singularity, and Lacia is depicted as a humanoid interface. In this story, human activities are monitored and predicted by AI as operating as infrastructure, and human agents “hack” the human mind socially through several human factors, including gender. “Beatless” is considered a key SF story for referenced in future AI design, with addressed the ethical problems associated with the introduction of AI into society, the ethical problems of persuasive engineering, as discussed by Fogg et al. (Fogg 1999), and gender difference problems in social factors, as explored by Nass et al. (Nass and Moon 2000) and corresponding cases were described experimentally. This provides a realistic example of the risks that interactive agent affective computing technology may present to decision-making (Picard 1997). These works will help the public to understand the pressing problems of information technology.

**6 Contribution and Limitations**.

This research has contributed guidelines for AI researchers on how to explain their work in the society. For instance, it is not appropriate to use SF related to human-like AI as an example of a system that operates as infrastructure for a connected society. In “Beatless,” for example, the question of who bears responsibility for decision-making in the decisions of infrastructuralized artificial intelligence is debated as an essential issue. When explaining similar social infrastructure AI, “Beatless” can be used to discuss the decision-making of infrastructure AI, showing that concerns based on human AI are not appropriate metaphors.

Our categorization also allows researchers to identify works that provide inspiring and simulative visions of AI. Important works are selected according to the criteria, and the results produce knowledge about stereotypes. By evaluating the artificial intelligence developed by engineers with the same parameters as these fictional portrayals, and using the results to classify their similarity to fictional AI, it is possible to address the problems described in the fictional as a possible virtual problem in advance. Based on the results of this survey, we believe it is appropriate to collect more extensive surveys from the general public using crowdsourcing and other methods.

The contribution of this research is to derive the range of contemporary popular imaginations of artificial intelligence and robots from sci-fi works based on the analysis of experts. Therefore, it is difficult at present to directly derive a future vision using SF alone. In the future, to understand through science fiction how AI fits into the various imaginary futures presented in literature and media, attention needs to be paid to the philosophical and empirical aspects of each work, as well as to the computation of narrative components. For example, what would it be like for people in the imaginary world to interact with AI or robotics and to live under the socio-technical conditions created thereby? Do patterns of human life continue to exist, what changes are likely, and what conditions exist? Is the story fundamentally optimistic about human nature and the ability to self-govern, or does it suggest that people need monitoring and guidance, and how does that human concept relate to the types of AI in the story, and how does it work? As a next step in this research, we believe that additional verification should be conducted from a multifaceted perspective, including literary scholars. Popular concerns about artificial intelligence technology can be addressed by separating the concerns that come from the literary visions of humans and tools from the real concerns that are extrapolated from real technology.

In a future work, we plan to use crowdsourcing to collect more data. The present work also involves a bias towards Japanese and American fiction. Science fiction from the United States has a strong influence on every country, including Japan. However, there is a risk that several results of this research may reflect a Japanese cultural background. It is possible that different trends may be observed in other countries. In the future, we plan to translate the questionnaire items into other languages and conduct international surveys. It should be noted that these analyses were correlative, not causal.

**7 Conclusion**.

We have surveyed and analyzed depictions of AI and robotic systems in the SF. As a result, stereotypes that AI researchers need to know in reference to science fiction have been identified, and areas that are important in communicating about future AI and robotics technologies to the public have been discovered. We also analyzed the contribution of several factors to the various vision of AI.

In this study, we hired critics and an author living in Japan with the help of a writer’s organization. Therefore, many of the selected works were limited to Japan or the United States, and most were novels. Many Japanese films are based on novels, which typically consider science from a multidisciplinary perspective. However, related research includes many studies on the influence of visual work, and we hope to extend future work to include more movies. The next step in this research is to develop a more detailed methods of communication. For example, the best fiction for conveying actual AI and robots to people can be selected by classifying actual AI according to the parameters of this study and searching for similar stories.

## Data Availability

The datasets generated during and/or analyzed during the current study are available from the corresponding author upon reasonable request.

## References

[CR1] Asimov I (1950). I, Robot.

[CR2] Asimov I (1978) “The Machine and The Robot.” in *Science Fiction: Contemporary Mythology*, edited by P. S. Warrick, M. H. Greenberg, and J. D. Olander. Harper and Row

[CR3] Brooks RA (1991) “Intelligence without Representation.” *Artificial Intelligence*

[CR4] Burton E, Goldsmith J, Mattei N (2018). How to Teach Computer Ethics through Science Fiction. Commun ACM.

[CR5] Cixin L, Translated by Gabriel Ascher (2013) Translated by Holger Nahm, and. “Beyond Narcissism: What Science Fiction Can Offer Literature.” *Science Fiction Studies* 40(1):22–32

[CR6] Ema A, Akiya N, Osawa H, Hattori H, Oie S, Ichise R, Kanzaki N, Kukita M, Saijo R, Otani T, Miyano N, Yoshimi Yashiro (2016). Future Relations between Humans and Artificial Intelligence: A Stakeholder Opinion Survey in Japan. IEEE Technol Soc Mag.

[CR7] Fogg BJ (1999). Persuasive Technologies. Commun ACM.

[CR8] Ganascia J-G (2010). Epistemology of AI Revisited in the Light of the Philosophy of Information. Knowl Technol Policy.

[CR9] Ganascia J-G (2017) *Intelligence Artificielle: Vers Une Domination Programmée ?*

[CR10] Haraway D (2000) “A Cyborg Manifest: Science, Technology, and Socialist-Feminism in the Late Twentieth Century. The cybercultures reader. Psychology Press, pp 291–324

[CR11] Inami M, Kawakami N, and Susumu Tachi (2003) Optical Camouflage Using Retro-Reflective Projection Technology. IEEE Computer Society

[CR12] Kaplan F (2004). Who Is Afraid of the Humanoid? Investigating Cultural Differences in the Acceptation of Robots. Int J Humanoid Rob.

[CR13] Kasahara S, Nagai S, Rekimoto J (2017). JackIn Head: Immersive Visual Telepresence System with Omnidirectional Wearable Camera. IEEE Trans Vis Comput Graph.

[CR14] Komatsu T, Kurosawa R (2012). How Does the Difference Between Users’ Expectations and Perceptions About a Robotic Agent Affect Their Behavior?. Int J Social Robot.

[CR15] Kurosu M (2014) “User Interfaces That Appeared in SciFi Movies and Their Reality.” Pp. 580–88 in *Design, User Experience, and Usability. Theories, Methods, and Tools for Designing the User Experience*, edited by A. Marcus. Cham: Springer International Publishing

[CR16] Leinweber DJ (2009). “Artificial Intelligence and Intelligence Amplification. Nerds on Wall Street.

[CR17] Marcus A, Sterling B, Swanwick M, Soloway E, and Vernor Vinge (1999) Opening Pleanary: Sci-Fi @ CHI-99: Science-Fiction Authors Predict Future User Interfaces. In Extended Abstracts on Human Factors in Computing Systems, 95–96. 10.1145/632716.63277

[CR18] Matsuo T (2017) “The Current Status of Japanese Robotics Law: Focusing on Automated Vehicles.” Pp. 151–70 in *Robotics, Autonomics and the Law*

[CR19] McCauley L (2007). AI Armageddon and the Three Laws of Robotics. Ethics Inf Technol.

[CR20] Mccauley L (2007) and Dunn Hall. “The Frankenstein Complex and Asimov’s Three Laws.” 9–14

[CR21] Mubin O, Billinghurst M, Obaid M, Jordan P, Alves-Oliveria P, Eriksson T, Barendregt W, Sjolle D, Fjeld M (2016) and Simeon Simoff. “Towards an Agenda for Sci-Fi Inspired HCI Research.” Pp. 1–6 in *Proceedings of the 13th International Conference on Advances in Computer Entertainment Technology*. New York, New York, USA: ACM Press

[CR22] Mubin O, Wadibhasme K, Jordan P (2019). Reflecting on the Presence of Science Fiction Robots in Computing Literature. ACM Trans Human-Robot Interact.

[CR23] Nagy P, Wylie R, Eschrich J, Finn Ed (2018). Why Frankenstein Is a Stigma Among Scientists. Sci Eng Ethics.

[CR24] Nass C (2000). Machines and Mindlessness: Social Responses to Computers. J Soc Issues.

[CR25] Nichols R, Smith ND (2007) and Fred Miller. “Philosophy Through Science Fiction: A Coursebook with Readings.” 448

[CR26] Omori N (2020) “Komatsu Sakyō: Japan’s Apocalyptic Sci-Fi Author in the Spotlight in 2020.” *Nippon.Com*. Retrieved (https://www.nippon.com/en/japan-topics/g00943/)

[CR27] Orikasa M, Inukai H, Eto K, Minamizawa K (2017) and Masahiko Inami. “Design of Sports Creation Workshop for Superhuman Sports.” Pp. 1–4 in *Proceedings of the Virtual Reality International Conference*. New York, New York, USA: ACM Press

[CR28] Picard RW (1997) Affective Computing. MIT Press

[CR29] Reeves S (2012) “Envisioning Ubiquitous Computing.” Pp. 1573–1582 in *Proceedings of the SIGCHI Conference on Human Factors in Computing Systems*, *CHI ’12*. New York, NY, USA: Association for Computing Machinery

[CR30] Rekimoto J (2014) “A New You: From Augmented Reality to Augmented Human.” Pp. 1–1 in *International Conference on Interactive Tabletops and Surfaces*. New York, New York, USA: ACM Press

[CR31] Robertson J (2011) “Gendering Robots:Posthuman Traditionalism in Japan.” Pp. 277–303 in *Recreating Japanese Men*

[CR32] Schmitz M, Endres C, Butz A (2008) A Survey of Human-Computer Interaction Design in Science Fiction Movies. Institute for Computer Sciences, Social-Informatics and Telecommunications Engineering

[CR33] Shaw-Garlock G (2009). Looking Forward to Sociable Robots. Int J Social Robot.

[CR34] Shedroff N (2012) and Chris Noessel. “Make It so: Learning from Sci-Fi Interfaces.” Pp. 7–8 in *International Working Conference on Advanced Visual Interfaces*. New York, New York, USA: ACM Press

[CR35] Sterling B (2009). “Design Fiction ” Interactions.

[CR36] Tanenbaum J, Tanenbaum K (2012) and Ron Wakkary. “Steampunk as Design Fiction.” Pp. 1583–1592 in *Conference on Human Factors in Computing System*, *CHI ’12*. New York, NY, USA: Association for Computing Machinery

[CR37] Tatsumi T (2000). Generations and Controversies: An Overview of Japanese Science Fiction, 1957–1997. Sci Fiction Stud.

[CR38] Troiano G, Tiab J, Youn-kyung Lim (2016) “SCI-FI: Shape-Changing Interfaces, Future Interactions.” Pp. 1–10 in *the 9th Nordic Conference*

[CR39] Vinge V (1993) “The Coming Technological Singularity: How to Survive in the Post-Human Era.&#8221

